# Elementary Ca^2+^ signals through endothelial TRPV4 channels regulate vascular function

**DOI:** 10.1186/2050-6511-14-S1-O23

**Published:** 2013-08-29

**Authors:** Mark T Nelson, Adrian D Bonev, Thomas Dalsgaard, Luis F Santana, John Scott, Michael I Kotlikoff, Swapnil K Sonkusare

**Affiliations:** 1 Department of Pharmacology, The University of Vermont, Burlington, VT, USA; 2 Department of Physiology, The University of Vermont, Burlington, VT, USA; 3 Pharmacology, University of Washington, Seattle, WA, USA; 4 Biomedical Sciences, Cornell University, Ithaca, NY, USA

## 

The endothelial cells (ECs) lining blood vessels are pivotal regulators of vascular tone. Endothelial-dependent vasodilation by classic agents such as acetylcholine (ACh) depends on an elevation of EC Ca^2+^, and can act through the generation of nitric oxide (NO). An elevation of EC Ca^2+^ also activates small- and intermediate-conductance (SK and IK) potassium channels. This leads to endothelium-dependent hyperpolarization (“EDH”), which is spread through gap junctions in specialized EC projections to adjacent smooth muscle cells (SMCs) to cause vasodilation of small resistance arteries and arterioles, and this pathway is responsible for the majority of the ACh-induced dilation in resistance arteries [1]. We have recently reported the first measurements of elementary Ca^2+^ influx events (“sparklets”) through single TRPV4 (transient receptor potential vanilloid 4) channels in ECs of intact, small mesenteric arteries. Cooperative opening of as few as 3 TRPV4 channels per EC caused maximum vasodilation primarily through activation of EC IK channels [2]. This raises the fundamental question about how these sparse channels maintain the functional linkages necessary for efficient signaling. An architectural feature of ECs that is likely centrally important in this regard is the myoendothelial projection (MEP). These specialized projections through the internal elastic lamina (IEL) connect ECs with adjacent SMCs through gap junctions. The objective of this study was to elucidate the signaling network that enables efficient and effective endothelial-dependent vasodilation through the EDH pathway. Our results indicate that muscarinic receptor agonists activate TRPV4 sparklets exclusively at MEPs in a protein kinase Cα (PKCα) and A-kinase anchoring protein (AKAP150)-dependent manner. We also found that elevation of extracellular K^+^ activates EC inward rectifier K^+^ (Kir) channels to cause vasodilation, and that TRPV4-mediated vasodilation is attenuated by block of Kir channels. Our results support the concept that AKAP150 localization to MEPs ensures the proximity of PKC to TRPV4 channels and enhances channel cooperativity. The resultant activation of MEP IK channels by TRPV4 sparklets causes a hyperpolarizing current as well as local accumulation of K^+^ ions in the “nanospace” between the MEP and SM membranes. This in turn could activate EC Kir channels to augment EDH. Compartmentalization of signaling elements by anchoring proteins at MEPs, along with a high-amplification network of ion channels at MEPs makes it possible for a small number of ion channels to exert a profound influence on vascular function independent of NO.
